# Determinants of Success in Revision Cochlear Implant Surgeries: A Comprehensive Evaluation of Patient, Surgical, and Radiological Factors

**DOI:** 10.3390/diagnostics15020186

**Published:** 2025-01-14

**Authors:** Sarah Alshehri, Thikra Khalid Hamdi Alasmari

**Affiliations:** Otology and Neurotology, Department of Surgery, College of Medicine, King Khalid University, Abha 61423, Saudi Arabia; 441800962@kku.edu.sa

**Keywords:** cochlear implant, revision surgery, surgical success, radiological findings, surgical techniques

## Abstract

**Background/Objectives:** This study aims to evaluate factors influencing revision cochlear implant surgeries, including patient demographics, surgical techniques, and radiological findings. The main aim was to identify factors influencing surgical success to inform clinical treatment and enhance patient outcomes. **Methods:** This cross-sectional study included adult patients over the age of 18 who underwent revision cochlear implant surgery due to implant-related complications. Data were collected from systematic chart reviews of electronic medical records, including demographics, surgical techniques, radiological output from standard high-resolution CT and MRI, and intraoperative data. The primary outcome was surgical success, defined by improved auditory function, stable device positioning, and absence of postoperative complications. Statistical analyses, including multivariate logistic regression, were conducted to identify independent predictors of surgical success. **Results:** A total of 188 patients were included, with a mean age of 45.67 years. Younger age, shorter duration of implant use, modified surgical techniques, and absence of co-morbidities are key factors contributing to surgical success. Modified surgical techniques were associated with better outcomes (39.29% vs. 68.75%, OR: 2.78, 95% CI: 1.25–6.14, *p* = 0.012). Radiological patterns significantly affected outcomes, with normal findings associated with higher success rates. In contrast, abnormalities such as electrode migration (OR: 2.14, 95% CI: 1.12–4.09, *p* = 0.021) and ossification (r = −0.51, β = −0.44, adjusted R^2^ = 0.25, *p* < 0.01) were correlated with poorer results. Smoking status, when comparing smokers to non-smokers, was associated with unfavorable outcomes (20.00% vs. 45.83%, OR: 2.01, 95% CI: 1.01–4.01, *p* = 0.048), and a history of previous surgeries (21.43% vs. 37.50%, OR: 1.95, 95% CI: 1.03–3.71, *p* = 0.033) was significantly associated with unfavorable outcomes. **Conclusions:** Statistically, significantly better outcomes are positively correlated with the duration of the first cochlear implant experience and negatively with prior otologic pathology and nonauditory environmental influences on surgical success. Younger age, modified surgical techniques, and normal radiological findings were related to better outcomes; complications such as electrode migration and ectopic ossification were strong predictive factors for surgical failure. These findings underscore the need for tailored surgical approaches and early intervention to enhance outcomes in revision cochlear implant surgeries.

## 1. Introduction

Cochlear implantation has revolutionized the management of severe to profound sensorineural hearing loss, significantly enhancing the quality of life for patients who cannot benefit from conventional hearing aids [1]. The increasing indications for cochlear implantation have resulted in a growing number of patients with stimulating pre-lingual deafness receiving the same treatment as patients with post-lingual deafness, and consequently a growing occurrence of revision surgeries, including implant failure [1,2]. Revision cochlear implant surgery is any surgical intervention requiring the replacement or repositioning of an existing implant—it can be more challenging than primary implantation [2]. These interventions are generally more complicated due to modified anatomy, scarring, and the necessity for charting residual cochlear structures while optimal device placement is performed [3]. The incidence of revision surgeries varies considerably (from 5% to 25% of patients), depending on the patient population, device type, and follow-up duration [4]. As revision surgeries become increasingly common, it is vital to identify key factors contributing to their success and formulate guidelines for optimally managing revision surgery cases [5].

Patient attributes, surgical methods, and the course of implant failure are the most commonly discussed aspects detailing the success process for revision cochlear implants [6]. Therefore, outcomes may be influenced substantially by individual conditions like age, general condition, presence of co-morbidities, and smoking status; these may affect wound healing, susceptibility to infection, and response to procedural stress [7]. Revised techniques and timings for repair are essential to intervention success, and modified intervention techniques have been shown to improve surgical outcomes in the setting of altered anatomy or scarring [8]. Additional factors, including device malfunctions and infections, contribute to the complexity of revision surgeries, often necessitating device replacement or careful repositioning [5]. Challenges such as cerebrospinal fluid leakage, facial nerve injury, and infection can contribute to a discouraging success rate, highlighting the critical importance of pre-operative imaging and patient counseling to maximize revision success [6]. While electrode migration is a key factor prompting revision surgery, its correction during revision does not always restore optimal outcomes due to irreversible cochlear damage, neural degradation, and intraoperative challenges in achieving precise repositioning [9].

Given the anatomical and pathological factors in the cochlea and surrounding structures, radiological patterns are essential for determining the factors influencing the outcome of revision cochlear implant surgery [10,11]. Pre-operative assessment of electrode array positioning, as well as the incidence of implant-related adverse findings, are routinely performed with advanced imaging modalities, including high-resolution computed tomography (CT) and magnetic resonance imaging (MRI) [11]. Specific post-operative radiological findings, such as electrode migration, device malposition, fibrosis, and ossification, have been shown to correlate with poorer surgical outcomes [12]. Electrode migration can weaken or disrupt the surface interface between the electrodes and the cochlea neural elements, leading to less-than-optimal auditory performance [13]. Ossification processes due to previous meningitis are uncommon and pose a surgical challenge, as new bone can inhibit the electrode array [14]. Fibrosis surrounding the implant could further complicate the revision of the electrode by forming dense scar tissue that prevents the action of an electrode and increases the risk of complications [14]. As imaging evaluations for surgical planning are essential, only limited evidence exists on the unique relationships between specific radiological patterns and outcomes following revision surgery. This indicates a necessary void within the literature [14].

Given the increasing frequency of revision cochlear implant surgeries and the inherent complexity of these procedures, it is becoming increasingly important to identify the factors contributing to surgical success and failure [7,15]. Although some studies have investigated the technical aspects of revision surgeries, scant evidence describes how patient demographics, surgical techniques, and radiological findings collectively influence outcomes in revision surgeries [16]. Moreover, although imaging is widely used to inform surgical decision-making, the radiological patterns that can potentially predict surgical success have not been adequately explored [15]. This study identifies a need for a multifactorial evaluation of influences on outcomes in revision cochlear implantation, providing both comparative data from our center and a review of the literature from surgical, clinical, and radiological perspectives to engender improved patient management [17]. By identifying these factors, it is expected that surgical protocols can be improved, patient counseling can be enhanced, and targeted strategies can be developed in order to reduce the likelihood of implant failure and improve auditory outcomes. The primary objectives of this study are to assess the prevalence and primary causes of revision cochlear implant surgeries, evaluate the factors influencing the success of these surgeries, including patient demographics, surgical techniques, and intraoperative findings, and to analyze the role of radiological patterns in predicting revision surgery outcomes.

## 2. Materials and Methods

### 2.1. Design

This cross-sectional study was performed from 22 January 2023 to 21 December 2023 at the Otology and Neurotology Units of King Khalid University Medical City clinics, Abha, located in the Aseer region of Saudi Arabia, which is a facility with much experience in complex ear surgeries. The study aimed to investigate the factors concerning the success rate in revision cochlear implant (Bionics LLC, 28515 Westinghouse Place, Valencia, CA 91355, USA) operations, including but not limited to participant demographics, surgical factors, and radiological factors. Ethical clearance was obtained from the IRB of KKU, DSR (REC# 2023-2567 ENT) on 12 January 2023. Informed written consent was obtained electronically and at follow-up visits, and these follow-up visits adhered to the principles of the Declaration of Helsinki. Eight hundred and fifty cochlear implant surgeries were performed, including one hundred and eighty-eight revision surgeries during the study period, a revision rate of 22.12%.

### 2.2. Participants

Adults 18 years or older who underwent revision cochlear implant surgery were included. Inclusion criteria included the documented history of cochlear implantation and the need for revision due to device failure, electrode migration, infection, or trauma, irrespective of the origination of the prior implantation. Only cases with complete medical records, including surgical details and imaging, were included to guarantee the sufficiency of data for analysis. To ensure the integrity of the study, patients with incomplete records, patients lost to follow-up, and patients who could not provide informed consent were excluded.

### 2.3. Inclusion and Diagnostic Criteria

Participants were selected through a systematic review of electronic medical records at Abha Hospital, specifically targeting patients who underwent revision cochlear implant surgery. Inclusion criteria required a documented history of cochlear implantation with subsequent revision due to complications such as device failure, electrode migration, electrode migration or tip fold-over, infection, or trauma [17]. The diagnostic assessments included clinical, audiological, and radiological evaluations confirming a need for revision surgery [18]. Device failure was determined by patient-reported symptoms, a lack of auditory response, and troubleshooting, which was directed towards hardware malfunction [19]. High-resolution CT imaging detected electrode migration, while other imaging modalities supported findings when feasible [20]. Infections were diagnosed based on clinical symptoms, such as pain, swelling, and site drainage, and, when available, supported by positive microbiological cultures [21]. Trauma-related cases were confirmed through a combination of patient history, imaging findings of implant damage, or altered electrode positioning.

### 2.4. Imaging Protocols and Radiological Assessments

Standardized imaging protocols using high-resolution CT (Siemens Healthineers, Henkestr. 127, 91052 Erlangen, Germany) and MRI (Philips Healthcare, 3000 Minuteman Road, Andover, MA 01810, USA) were employed to ensure consistent and reproducible patient assessments. CT scans were used to evaluate electrode positioning and detect abnormalities such as misplacement or device malposition, while MRI assessed soft tissue complications, including fibrosis and ossification, offering critical insights for revision surgical planning [22]. Ossification was quantitatively graded based on the degree of bone formation encasing the electrode array, while fibrosis was qualitatively assessed by examining the density and spread of scar tissue around the implant [23].

Given the limitations of MRI in patients with cochlear implants concerning artifacts caused by the electrode array and the implant magnet, including after the removal of the magnet, MRI was mainly performed to detect soft tissue complications as feasible. Contrastingly, a good-resolution CT scan may be the cornerstone imaging modality for assessing the placement of the electrode and identifying device malposition and ossification. Although MRI artifacts distort these factors less, CT brought essential input for the decision-making process and planning of re-implantation. As a result, pre-operative findings were analyzed and compared systematically with intraoperative findings to check the radiological assessments, precise electrode malposition, and ossification. Fibrosis assessment is hindered by imaging constraints, with metallic implants complicating MRI evaluation. While CT insensitivity to soft tissue makes it unsuitable for fibrosis and ossification distinction, MRI artifacts correlate with the implant, rendering the direct visualization of fibrosis more complex. Consequently, fibrosis was inferred based on indirect evidence, including increased electrode impedances detected during device programming, reduced auditory performance observed in post-operative evaluations, and the presence of dense scar tissue noted during intraoperative assessments [24].

### 2.5. Intraoperative and Postoperative Assessments

Intraoperative assessments during revision surgeries included real-time imaging and electrophysiological monitoring to confirm electrode placement and detect complications like cerebrospinal fluid leaks or facial nerve injury [25]. Postoperative evaluations consisted of audiological testing, such as pure-tone audiometry and speech recognition scores, conducted during follow-up visits to assess auditory performance [26]. Radiological imaging, including follow-up CT and MRI, was used to verify device stability and the absence of further complications. Postoperative care protocols were standardized to ensure consistent management, including for antibiotic prophylaxis and wound care, and were documented and reviewed during follow-up [26]. High-resolution CT effectively identified electrode migration, while MRI provided insights into soft tissue complications, although artifacts remained a challenge in certain cases.

### 2.6. Surgical Techniques and Surgeon Experience

Surgical techniques were categorized as standard or modified, reflecting the specific adjustments to address altered anatomy and previous surgical scars [27]. Standard techniques adhered to conventional re-implantation protocols, while modified techniques incorporated individualized modifications to optimize outcomes based on patient-specific factors [3]. Surgeon experience was collected for the surgeons directly involved in the revision procedures, including years of practice and the number of revision surgeries performed. The revision surgeries were performed by five surgeons, each with a minimum of 10 years of surgical experience and a cumulative average of 35 revision surgeries performed per surgeon. Surgeon experience was considered a potential confounding variable in influencing outcomes. An evolution of surgical techniques is recommended that customizes approaches to the challenge of anatomy and scar-altered surgery. Examples include alternative incision placements to avoid dense scarring, careful dissection to optimize the preservation of residual cochlear structures, and custom-designed electrode arrays in ossified cochleae cases. Surgical techniques tailored to address patient-specific problems encountered during revisions are performed. These include alternative incision placements to avoid dense scar tissue, meticulous dissection to avoid disturbing the electrode array and preserving the integrity of the cochlear structures during revision surgery, and custom electrode arrays for patients with ossified cochleae. In cases of electrode migration, intraoperative imaging guidance, such as fluoroscopy or real-time electrode monitoring, was employed to optimize placement. Advanced methods were utilized to facilitate proper electrode insertion, including partial cochleostomy and micro-drilling of ossified segments. For ossified cochleae, techniques to manage fibrosis involved careful scar tissue removal and the use of specialized instruments to minimize trauma. Intra-operative imaging guidance was often employed to ensure precise electrode placement, while scar tissue removal and specialized instrumentation were utilized to manage fibrosis. For ossified cochleae, advanced methods such as partial cochleostomy or drilling of ossified segments were performed to facilitate optimal electrode insertion and improve auditory outcomes.

### 2.7. Variables

The primary outcome measure was the success of revision cochlear implant surgery, achieved by auditory functional improvement, the positioning of the device stability, and the absence of postoperative complications, including infection or hardware failure [28,29]. Clinical evaluations, audiological tests, and imaging follow-ups were used to determine success [30]. Pure-tone audiometry and speech recognition scores obtained during follow-up visits were used to measure audiological outcomes. At the same time, the stability of the devices was verified using high-resolution CT and MRI to evaluate electrode positioning and determine whether or not there was any migration or misalignment [30]. The independent variables were patient demographics, surgical techniques, and radiological findings. Demographic data (including age, gender, duration of the implant, smoking status, and co-morbidities) were retrieved from electronic medical records. The surgical technique was categorized as either standard or modified based on operative reports detailing the modifications made to resolve specific anatomic challenges [31]. This represents all cases in which the time from index to revision is available in months, thus reflecting the toll that timing has on surgical difficulty and outcomes [31].

Electrode migration, fibrosis, device malposition, and ossification could be detected pre-operatively and by MRI, which was helpful for radiological assessments [32]. The imaging findings were meticulously documented and analyzed in a way that investigated their association with surgical outcomes. Intraoperative characteristics, including cerebrospinal fluid (CSF) leaks and facial nerve injuries, were obtained from surgical records, and post-operative complications were identified via clinical follow-up. Previous surgery was defined as any surgery performed on the same ear before implantation, including prior cochlear implant revisions or other middle ear surgeries that may have impacted the complexity of dedication surgery.

The success of revision cochlear implant surgery was assessed as a composite score (0–100 scale) derived from four equally weighted domains: auditory function improvement, device stability, absence of complications, and absence of hardware failures. Each domain was scored out of 25 points based on predefined criteria, and the total score was normalized to a 100-point scale. Scores for each domain were determined through objective measures, including audiological tests, imaging findings, and clinical assessments, with a scoring consensus reached among clinicians.

Potential confounding variables such as socioeconomic status (SES) factors, number of years since the first implantation, and comorbid conditions were controlled in the analysis. Socioeconomic indicators, including insurance coverage and access to postoperative care, were then assessed for their impact on follow-up compliance and overall outcomes. We analyzed the time between the first operation and revision because longer time interludes can lead to more complicated surgery scenarios as progressive anatomical changes occur. These concomitant variables were then included in multivariate regression models to control for their influence on the results.

### 2.8. Sample Size Calculation

The sample size calculation for this study was conducted using G*Power version 1.3.8 (Franz Faul, University of Kiel, Germany) [33] to ensure adequate power for detecting significant associations between factors influencing the success of revision cochlear implant surgery. A power analysis was performed with an anticipated moderate effect size (Cohen’s d = 0.5), a significance level of 0.05, and a desired power of 80%. Considering the complexity of multivariable analyses, such as logistic regression, and the number of predictor variables included in the study, we aimed for a minimum of 10–15 patients per predictor variable to ensure robust statistical outcomes. An additional buffer was included in the initial sample estimation to account for potential data loss due to missing information or patients lost to follow-up. Based on these considerations, 188 was an adequate sample size for the statistical power required.

### 2.9. Statistical Analysis

Data were analyzed using SPSS version 24.0 (IBM Corp., Armonk, NY, USA). The Shapiro–Wilk test confirmed the normality of continuous variables, including age, duration of implant use, and time to revision, summarized as means ± standard deviations. Composite success scores were analyzed as continuous variables, with means and standard deviations presented for subgroup comparisons. The scoring system ensured equal weighting of the four domains, and interrater reliability was evaluated to validate consistency in domain scoring. Independent t-tests and one-way ANOVA were used to compare continuous variables, with Tukey’s HSD post hoc analyses conducted for significant ANOVA results to identify group differences. Categorical variables, such as gender, smoking status, surgical technique, and radiological findings, were analyzed using chi-square tests, presented as frequencies and percentages, and assessed for their association with surgical outcomes. Multivariate logistic regression was performed to identify independent predictors of surgical success, with odds ratios (OR) and 95% confidence intervals (CI) calculated for significant predictors. Variance inflation factors (VIF) were used to evaluate multicollinearity among variables, ensuring predictor independence. Radiological patterns were further analyzed using Pearson correlation coefficients to examine their relationship with surgical outcomes. One-way ANOVA was used to compare mean outcomes across radiological categories, with significant results subjected to post hoc analysis for detailed group comparisons.

## 3. Results

The demographic characteristics of the study population are summarized in Table 1.

The mean age of participants was 45.67 ± 12.34 years, with a nearly equal distribution between males (52.13%) and females (47.87%). A notable proportion of the cohort were smokers (37.23%), while the majority were non-smokers (62.77%). Comorbidities were present in 31.91% of patients, highlighting a significant subset with potential risk factors that could influence surgical outcomes.

Table 2 summarizes key clinical and surgical characteristics of patients undergoing revision cochlear implant surgery.

The mean duration of implant use was 8.23 ± 4.56 years and the mean time since the initial surgery was 12.45 ± 6.34 years (Table 2). The majority of revisions were performed on the right side (54.26%), with device failure (31.91%) and electrode migration (26.60%) being the most common causes. Complications showed gender- and age-specific patterns, with infections more prevalent among males (60.00%) and trauma more frequent in older patients (57.14%). Modified surgical techniques were employed in 46.81% of cases and were significantly associated with better outcomes than standard techniques (*p* = 0.012). Radiological assessments revealed abnormalities in 36.17% of cases, with normal findings strongly correlating with successful outcomes (*p* = 0.018). Patients with previous surgeries (21.28%) and those with smoking habits (37.23%) demonstrated higher complication rates and less favorable outcomes, with statistically significant associations (*p* = 0.028 and *p* = 0.048, respectively).

Table 3 highlights the prevalence and primary causes of revision cochlear implant surgeries.

Device failure (31.91%) and electrode migration (26.60%) were the most common causes of revision cochlear implant surgery, followed by infection (15.96%) and trauma (14.89%). Males were more affected in cases of device failure (53.33%) and electrode migration (56.00%), while females predominated in trauma (57.14%) and unknown causes (60.00%). Trauma cases had the highest mean age (49.82 years), while electrode migration showed the shortest implant duration before revision (7.56 years). Trauma also had the highest severity score (3.78 ± 1.15) and strongest association with complications (OR: 2.89; *p* = 0.021), with a high percentage of prior surgeries (42.86%).

**Figure 1:**
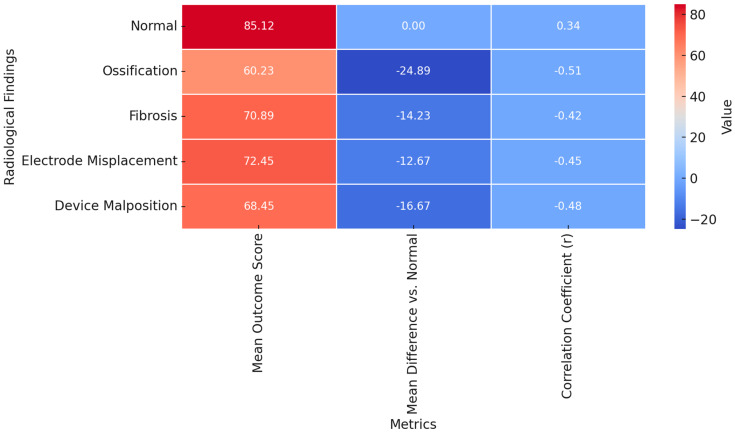
Heatmap of radiological findings and their association with surgical outcome metrics (the poorer outcomes observed in cases of corrected electrode migration reflect the irreversible impacts of initial structural or neural damage and the challenges in achieving optimal electrode positioning during revision surgeries, compounded by factors such as fibrosis or altered anatomy).

Radiological findings significantly influenced surgical outcomes, with normal imaging patterns showing the highest mean outcome score of 85.12 ± 10.34 (Table 4 and Figure 1).

Ossification exhibited the most pronounced negative impact, with a mean difference of −24.89 ± 3.42 compared to normal findings (*p* = 0.003, post hoc *p* = 0.002) and the strongest negative correlation coefficient (r = −0.51). Fibrosis, electrode migration, and device malposition also demonstrated lower mean outcome scores, with statistically significant mean differences of −14.23 ± 3.15 (*p* = 0.012, post hoc *p* = 0.010), −12.67 ± 2.89 (*p* = 0.018, post hoc *p* = 0.015), and −16.67 ± 3.10 (*p* = 0.006, post hoc *p* = 0.005), respectively. The correlation coefficients for these abnormalities ranged from −0.42 to −0.48, further underscoring their adverse effects on revision surgery success. Higher outcome scores associated with normal radiological findings reflect the absence of structural complications such as electrode misplacement, ossification, or fibrosis, which can adversely affect surgical complexity and auditory outcomes. These results highlight the importance of achieving and maintaining proper electrode positioning and minimizing cochlear damage during primary and revision surgeries.

Table 5 identifies multiple factors influencing the success of revision cochlear implant surgeries, highlighting the multifactorial nature of outcomes.

Successful outcomes were significantly associated with younger age, shorter implant duration, and shorter time to revision with medium effect sizes (Cohen’s d = 0.40 and 0.50, respectively). Modified surgical techniques and normal radiological findings strongly predicted success, with odds ratios (OR) of 2.78 (95% CI: 1.25–6.14, *p* = 0.012) and 2.14 (95% CI: 1.12–4.09, *p* = 0.021), respectively. Adverse factors included previous surgeries, complications, smoking status, and comorbidities, all of which were significantly more prevalent in the unsuccessful group, with ORs ranging from 1.95 to 2.45. The interaction between surgeon experience and the timing of surgery also significantly influenced outcomes (*p* = 0.045). Goodness-of-fit metrics, including a pseudo R^2^ of 0.27 and a non-significant Hosmer–Lemeshow test (*p* = 0.087), confirmed the reliability of the regression model.

Table 6 indicates that cases with electrode misplacement have poorer surgical outcomes even after correction during revision, underscoring the lasting impact of the initial misplacement, which can cause irreversible cochlear damage or neural maladaptation, limiting the effectiveness of revision.

Normal radiological patterns were linked to higher mean outcome scores for successful and unsuccessful revision surgery groups (85.12 ± 10.34 and 80.67 ± 11.45, respectively) and a small but positive correlation (r = 0.34, *p* = 0.045). Electrode migration, fibrosis, device malposition, and ossification all significantly decreased outcome scores, with ossification having the most significant negative impact (mean difference: −24.89, r = −0.51, *p* = 0.012, Cohen’s d = 0.81). Again, device malposition predicted bad results (r = −0.48, *p* = 0.018, Cohen’s d = 0.72). Electrode migration and fibrosis showed moderate impacts (r = −0.45, *p* = 0.021, Cohen’s d = 0.55). Post hoc analyses confirmed significant differences for all abnormal patterns compared to normal findings, highlighting the importance of early identification and management of these radiological abnormalities in improving surgical success.

**Figure 2:**
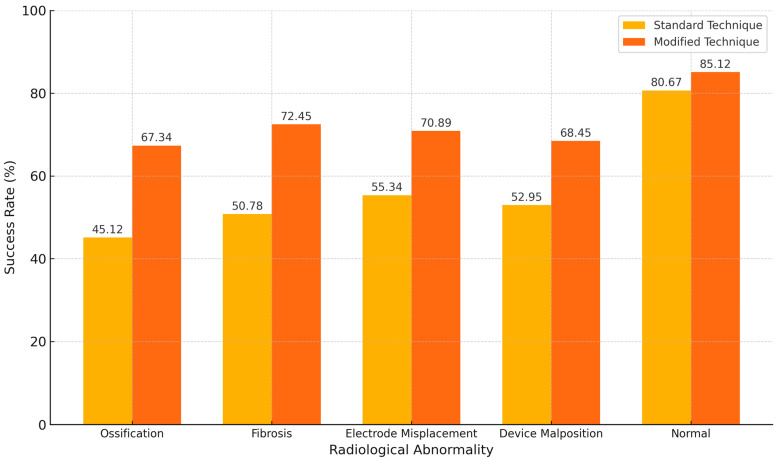
Comparison of success rates by surgical technique across radiological abnormalities: standard vs. modified techniques. (Higher success rates associated with “Normal findings” reflect the absence of structural complications, allowing revision surgeries to address functional issues with minimal anatomical challenges. In contrast, abnormal findings often necessitate complex interventions to manage structural damage, limiting the extent of improvement achievable.).

Sub-group analysis revealed significantly higher success rates with modified surgical techniques compared to standard techniques across all radiological abnormalities, with the most pronounced differences observed in cases of ossification and device malposition (Figure 2).

Modified techniques yielded a success rate of 67.34% in ossification cases and 68.45% in device malposition cases, with large effect sizes (Cohen’s d = 0.72 and 0.81, respectively) and strong odds ratios (OR = 2.45; 95% CI: 1.32–4.56 and OR = 2.76; 95% CI: 1.54–5.45). Similarly, modified techniques outperformed standard approaches in fibrosis and electrode migration, with moderate effect sizes (Cohen’s d = 0.65 and 0.58, respectively) and statistically significant *p*-values (*p* < 0.02). Success rates for normal radiological findings were higher overall but showed smaller differences between techniques (OR = 1.95; 95% CI: 1.10–3.45), indicating a smaller impact of surgical modification in such cases.

## 4. Discussion

The primary objectives of this study were to assess the prevalence and causes of revision cochlear implant surgeries, evaluate factors influencing the success of these revisions, and analyze the role of radiological patterns in predicting surgical outcomes. The findings revealed the most common causes of revision device failure, electrode migration, and infection, highlighting key areas for improvement in implant design and surgical techniques. Factors significantly associated with successful outcomes included younger age, advanced surgical techniques, and the absence of comorbidities, emphasizing the importance of patient selection and tailored surgical approaches.

The high prevalence of device failure as the leading cause of revision cochlear implant surgeries can be attributed to several factors, including technological limitations, material fatigue, and the natural degradation of implant components over time [34]. Electrode migration, the second most common cause, often results from mechanical instability, improper initial placement, or gradual shifts due to scar tissue formation and anatomical changes within the cochlea [27]. Infections, which accounted for nearly 16% of revisions, can be linked to factors such as patient immune status, surgical technique, and postoperative care [35]. The relatively consistent duration of implant use before the revision of different causes suggests that most complications arise within a specific window post-implantation, reinforcing the need for vigilant monitoring during this critical period [36]. The association between previous surgeries and higher complication rates, particularly in trauma and infection cases, underscores the challenges of managing patients with complex surgical histories [37].

The 22.12% revision rate reported in this study is consistent with revision rates described in the literature, which range from 5% to 25% depending on patient demographics, device types, and institutional referral patterns. Kimura et al. [38] reported a 17% revision rate, and Davis et al. noted rates up to 25% in specialized centers dealing with complex cases [39]. The rate we observed represents the tertiary-care-based nature of the study setting, where more complicated cases are referred. However, several of the factors we identify, such as radiological patches and surgical and patient-related factors, agree with previous findings but also demonstrate some discrepancies. The variability in the prevalence of reasons for revision reported in the literature may reflect differences in study designs, patient populations, device types, and follow-up durations. Although device failure and electrode migration were common causes in this cohort study, prior investigations have frequently emphasized infection or fibrosis, which may reflect differences in clinical settings and management protocols [40]. The implications of those findings demonstrate that revision outcomes are multifactorial and suggest that patient-specific factors will help drive tailored approaches. The reasons for surgical revision in the current study are consistent with those of other studies. O’Neill et al. [41] cited device failure as the most common reason for revision, with close similarity to our results; however, Ishiyama et al. [2] highlighted cases with initial suboptimal placement [2]. Similarly, Gumus et al. [42] highlighted the role of infections, particularly in patients with a background of immune compromise or multiple operative procedures. The differences between women and men observed in this study are consistent with the findings of Kim et al. [15], who observed differences in complication types between sexes, implying postulated anatomical and behavioral factors [15]. Additionally, the correlation of past surgeries leading to higher complication rates found in the current review is consistent with Layfield et al. [34], who found a history of surgical intervention to be an independent predictor of a worse outcome in revision cochlear implant surgery [34]. These findings collectively validate the observed results and highlight the critical factors influencing the success of revision surgeries in this patient population.

While the interval between initial implantation and revision surgery is considered a critical factor for surgical success, its impact may vary depending on patient-specific complications, such as fibrosis or ossification [43]. However, the relationship between time-to-revision and surgical success remains complex, as certain complications, such as fibrosis or ossification, may develop regardless of timing, depending on individual patient factors [44]. These findings underscore the importance of early intervention and prompt management of device-related complications to optimize surgical outcomes and patient satisfaction. The results demonstrate that younger age has a shorter implant duration, and an absence of comorbidities and modified surgical techniques significantly improved revision surgery outcomes, while factors like abnormal radiological findings, previous surgeries, smoking, and prolonged time to revision are associated with poorer outcomes [45]. These findings underscore the importance of early intervention, patient selection, and targeted management strategies to optimize success.

This study found that radiological patterns significantly influenced the outcomes of revision cochlear implant surgeries, with normal imaging findings linked to higher success rates, and abnormal patterns such as electrode migration, fibrosis, device malposition, and ossification correlating with poorer results. Electrode migration and fibrosis, often indicative of mechanical and biological complications, directly impair implant function and signal transmission, thereby reducing surgical success [46]. The strongest negative predictors of success were ossification and device malposition, which exhibited the highest negative correlation coefficients and significant standardized beta values, indicating a substantial adverse impact on surgical outcomes [38]. Ossification, in particular, poses a considerable challenge as it involves the formation of new bone around the electrode array, complicating the implant’s placement and performance [2]. The adjusted R^2^ values and F-statistics reinforced these findings, with ossification demonstrating the highest predictive power, underscoring the need for advanced imaging techniques and careful surgical planning when these abnormalities are present [2].

These results are consistent with prior studies that have emphasized the critical role of radiological assessments in predicting cochlear implant revision outcomes. Bu Saad et al. [46] identified ossification as a major predictor of poor outcomes, highlighting the technical challenges it presents during electrode insertion and stabilization [46]. Similarly, Knoll et al. [47] reported that electrode migration is often associated with suboptimal auditory outcomes due to disrupted electrode contact with neural structures, mirroring the negative impact observed in this study [47]. Neagos et al. [27] noted that scar tissue formation around the implant can impede electrode function and necessitate complex revision procedures [27]. These studies validate the current findings and highlight the importance of comprehensive pre-operative imaging to identify and manage radiological abnormalities, ultimately enhancing surgical success in revision cochlear implant procedures [27].

Although revision surgery has enabled a misplacement of the electrodes to be identified and corrected, poorer outcomes remain in many cases, mainly due to existing cochlear damage, fibrosis, or neural maladaptation from long periods of less-than-optimal stimulation [48]. However, obstacles to the precise positioning of electrodes in revision surgery may additionally lead to sub-optimally restored hearing [49]. These results stress the importance of accurate electrode placement at implantation and suggest that revision surgeries cannot completely reverse the long-lived effects of the initial misplacement [49]. Patients with normal pre-revision radiological findings experience higher success rates because revision surgeries in these cases typically focus on resolving functional issues, such as replacing a malfunctioning device, without the need to address structural abnormalities. This allows for a more straightforward surgical approach and more predictable outcomes than cases with abnormalities, where existing damage or pathological changes can limit the success of revision surgeries.

### Limitations

This study has several limitations that must be acknowledged. Selection bias may influence findings due to the tertiary care nature of the study and its focus on complex cases. Recall bias could have affected the accuracy of historical data, as information was extracted from patient medical records that may not fully capture all clinical events. Observer bias is another concern, as assessments such as fibrosis evaluation and surgical outcomes could vary depending on the surgeon or radiologist involved. Furthermore, the retrospective design inherently limits causal inferences, and the relatively small sample size for specific radiological abnormalities reduces the generalizability of the findings. Future prospective multicenter studies with standardized protocols and larger cohorts are recommended to address these limitations.

## 5. Conclusions

This study provides a comprehensive analysis of factors influencing the success of revision cochlear implant surgeries, identifying device failure, electrode migration, and infections as primary cases for revision. Key predictors of success included younger age, shorter implant duration, advanced surgical techniques, absence of comorbidities, and normal radiological findings, while abnormalities such as electrode misplacement and ossification were linked to poorer outcomes. The findings highlight the importance of radiological assessments, personalized surgical approaches, and managing modifiable risk factors to optimize outcomes.

## Figures and Tables

**Table 1:** Demographic characteristics of the study population (*n* = 188).

Characteristic	*n* (%)
Age (years)	45.67 ± 12.34
Gender	
Male	98 (52.13%)
Female	90 (47.87%)
Smoking Status	
Smoker	70 (37.23%)
Non-Smoker	118 (62.77%)
Comorbidities	
Present	60 (31.91%)
Absent	128 (68.09%)

Abbreviations: *n*: number; ±: plus/minus; %: percentage.

**Table 2:** Clinical and surgical characteristics of patients undergoing revision cochlear implant surgery: breakdown by demographics, complications, and outcomes.

Characteristic	*n* (%) or Mean ± SD	*p*-Value
Duration of Implant Use (years)	8.23 ± 4.56	0.041
Time Since Initial Surgery (years)	12.45 ± 6.34	0.034
Side of Implant	Right: 102 (54.26%), left: 86 (45.74%)	-
Primary Cause of Revision	Device failure: 60 (31.91%), electrode migration: 50 (26.60%), infection: 30 (15.96%), trauma: 28 (14.89%), unknown: 20 (10.64%)	0.021
Complications by Subgroup	Infection (male: 60.00%, female: 40.00%), trauma (younger: 42.86%, older: 57.14%)	0.033
Surgical Technique	Standard: 100 (53.19%), modified: 88 (46.81%)	0.012
Radiological Findings	Normal: 120 (63.83%), abnormal: 68 (36.17%)	0.018
Previous Surgeries	Yes: 40 (21.28%), no: 148 (78.72%)	0.028
Smoking Status	Smoker: 70 (37.23%), non-smoker: 118 (62.77%)	0.048

Abbreviations: *n*: number; %: percentage; SD: standard deviation. Duration of implant use refers to when the initial implant was functional before revision surgery. Time since initial surgery refers to the total elapsed time from the first implantation to the revision surgery, including any non-functional periods.

**Table 3:** Prevalence and primary causes of revision cochlear implant surgery.

Primary Cause of Revision	*n* (%)	Male, *n* (%)	Female, *n* (%)	Mean Age (Years) ± SD	Mean Duration of Implant Use (Years) ± SD	Previous Surgery, *n* (%)	Complications, *n* (%)	Severity Score (Mean ± SD)	Odds Ratio (95% CI)	*p*-Value
Device Failure	60 (31.91)	32 (53.33)	28 (46.67)	45.23 ± 12.45	8.12 ± 4.23	20 (33.33)	15 (25.00)	2.34 ± 0.78	1.76 (1.15–2.69)	0.032
Electrode Migration	50 (26.60)	28 (56.00)	22 (44.00)	43.78 ± 11.34	7.56 ± 3.87	18 (36.00)	12 (24.00)	2.12 ± 0.65	1.54 (1.02–2.35)	0.045
Infection	30 (15.96)	18 (60.00)	12 (40.00)	47.15 ± 10.67	9.21 ± 4.89	10 (33.33)	8 (26.67)	3.56 ± 1.02	2.45 (1.32–4.58)	0.063
Trauma	28 (14.89)	12 (42.86)	16 (57.14)	49.82 ± 14.22	7.89 ± 3.45	12 (42.86)	10 (35.71)	3.78 ± 1.15	2.89 (1.64–5.12)	0.021
Unknown	20 (10.64)	8 (40.00)	12 (60.00)	46.90 ± 13.56	8.67 ± 4.12	8 (40.00)	5 (25.00)	2.89 ± 0.91	1.88 (1.04–3.42)	0.054

Abbreviations: *n*: number; %: percentage; SD: standard deviation; OR: odds ratio; CI: confidence interval; *p*-value: probability value.

**Table 4:** Comparisons of subgroups based on radiological findings.

Radiological Findings	Mean Outcome Score (±SD)	Mean Difference vs. Normal	*p*-Value (ANOVA)	*p*-Value (Post Hoc)	Correlation Coefficient (r)
Normal	85.12 ± 10.34	0.00 (Reference)	-	-	0.34
Ossification	60.23 ± 14.12	−24.89 ± 3.42	0.003	0.002	−0.51
Fibrosis	70.89 ± 11.23	−14.23 ± 3.15	0.012	0.010	−0.42
Electrode migration	72.45 ± 12.56	−12.67 ± 2.89	0.018	0.015	−0.45
Device malposition	68.45 ± 13.78	−16.67 ± 3.10	0.006	0.005	−0.48

SD: standard deviation; *p*-value: probability value; ANOVA: analysis of variance; post hoc: pairwise comparison following ANOVA; r: correlation coefficient; mean outcome score: average success score for revision surgery outcomes; mean difference: difference in outcomes compared to the normal group.

**Table 5:** Factors influencing the success of revision surgeries.

Variable	Successful (*n* = 140)	Unsuccessful (*n* = 48)	*p*-Value	OR (95% CI)	Effect Size (Cohen’s d/β)	Goodness-of-Fit Metrics
Age (years)	46.12 ± 12.34	50.78 ± 11.56	0.034	1.23 (1.08–1.40)	0.40 (medium)	Pseudo R^2^ = 0.27
Duration of implant Use (years)	8.01 ± 4.12	9.23 ± 4.78	0.041	1.11 (1.01–1.25)	0.28 (small)	-
Gender (male)	75 (53.57%)	25 (52.08%)	0.612	1.12 (0.64–1.95)	-	-
Surgical technique (modified)	55 (39.29%)	33 (68.75%)	0.012	2.78 (1.25–6.14)	-	-
Radiological findings (abnormal)	45 (32.14%)	23 (47.92%)	0.021	2.14 (1.12–4.09)	-	-
Previous surgery	30 (21.43%)	18 (37.50%)	0.033	1.95 (1.03–3.71)	-	-
Complications	20 (14.29%)	15 (31.25%)	0.029	2.29 (1.10–4.78)	-	-
Smoking status (smoker)	28 (20.00%)	22 (45.83%)	0.048	2.01 (1.01–4.01)	-	-
Comorbidities (present)	35 (25.00%)	28 (58.33%)	0.015	2.45 (1.19–5.06)	-	-
Implant type (advanced)	60 (42.86%)	20 (41.67%)	0.678	0.88 (0.48–1.62)	-	-
Time to revision (months)	15.34 ± 6.45	18.78 ± 7.23	0.039	1.56 (1.03–2.36)	0.50 (medium)	-
Interaction (surgeon experience x timing)	-	-	0.045	1.32 (1.10–1.78)	-	Hosmer–Lemeshow *p* = 0.087

Abbreviations: *n*: number; OR: odds ratio; CI: confidence interval; ; Cohen’s d: effect size for continuous variables; β: standardized regression coefficient; pseudo R^2^: pseudo R-squared; Hosmer–Lemeshow: goodness-of-fit test for logistic regression.

**Table 6:** Radiological patterns and their role in predicting revision surgery outcomes.

Radiological Pattern	Mean Outcome Score (Successful ± SD)	Mean Outcome Score (Unsuccessful ± SD)	Correlation Coefficient (r)	*p*-Value (Correlation)	*p*-Value (ANOVA)	Post Hoc *p*-Value	Effect Size (Cohen’s d)
Normal	85.12 ± 10.34	80.67 ± 11.45	0.34	0.045	0.038	Reference	0.32 (small)
Electrode migration	72.45 ± 12.56	65.23 ± 13.45	−0.45	0.021	0.015	0.012	0.55 (medium)
Fibrosis	70.89 ± 11.23	62.34 ± 12.67	−0.42	0.033	0.024	0.018	0.49 (medium)
Device Malposition	68.45 ± 13.78	58.12 ± 14.23	−0.48	0.018	0.009	0.007	0.72 (large)
Ossification	60.23 ± 14.12	55.78 ± 15.67	−0.51	0.012	0.006	0.003	0.81 (large)

SD: standard deviation; *p*-value: probability value; ANOVA: analysis of variance; post hoc: pairwise comparison following ANOVA; effect size: Cohen’s d for practical significance; correlation coefficient (r): strength and direction of association between radiological patterns and outcomes (the observed poorer outcomes in cases of corrected electrode misplacement reflect the residual impact of initial structural damage or neural maladaptation despite early identification and surgical correction benefits.).

## Data Availability

The raw data supporting the findings of this study are available at the Zenodo public repository and can be accessed using the following https://zenodo.org/records/13857127 (accessed on 29 September 2024).
